# Elucidating the molecular docking and binding dynamics of aptamers with spike proteins across SARS-CoV-2 variants of concern

**DOI:** 10.3389/fmicb.2025.1503890

**Published:** 2025-02-14

**Authors:** Irwin A. Quintela, Tyler Vasse, Dana Jian, Cameron Harrington, Wesley Sien, Vivian C. H. Wu

**Affiliations:** Produce Safety and Microbiology Research Unit, U.S. Department of Agriculture, Agricultural Research Service, Western Regional Research Center, Albany, CA, United States

**Keywords:** aptamers, SARS-CoV-2, ELONA, spike proteins, SELEX (systematic evolution of ligands by exponential enrichment)

## Abstract

DNA aptamers with high binding affinity against SARS-CoV-2 spike proteins have been selected and analyzed. To better understand the binding affinities between DNA aptamers and spike proteins (S-proteins) of relevant variants of concerns (VOCs), *in silico* and *in vitro* characterization are excellent approaches to implement. Here, we identified and generated DNA aptamer sequences targeting the S-protein of SARS-CoV-2 VOCs through systematic evolution of ligands by exponential enrichment (SELEX). *In silico*, prediction of aptamer binding was conducted, followed by a step-by-step workflow for secondary and tertiary aptamer structures determination, modeling, and molecular docking to target S-protein. The *in silico* strategy was limited to only providing predictions of possible outcomes based on scores, and ranking was complemented by characterization and analysis of identified DNA aptamers using a direct enzyme-linked oligonucleotides assay (ELONA), which showed dissociation constants (*K*_d_) within the 32 nM–193 nM range across the three significant VOCs. These three highly specific VOCs aptamers (Alpha Apt, Delta Apt, and Omicron Apt) can be further studied as potential candidates for both diagnostic and therapeutic applications.

## 1 Introduction

Since the first appearance of SARS-CoV-2 in late 2019, its variants have been classified into key groups—such as variants of concern (VOCs), variants of interest (VOIs), variants being monitored (VBMs), and variants of high consequence (VOHCs)—based on predicted virulence, transmissibility, and disease severity (Marino et al., 2020; Jung et al., 2024; Nesamari et al., 2024). Specifically, Alpha, Delta, and Omicron VOCs were highly successful variants that resulted in sequential viral strain replacements over time (Meganck et al., 2024). SARS-CoV-2 infection is initiated by virus interaction with host’s ACE2 cell-surface receptors and proteases, which activates the spike (S) proteins, followed by the fusion of viral and cellular membranes to release the virus genome into the host cell (Pizzato et al., 2022; Steiner et al., 2024). These initial interaction and fusion activities are mediated by SARS-COV-2 spike glycoprotein, which has become a primary target of vaccine development, diagnostic, and therapy (Lucena et al., 2024; Prabhakaran et al., 2024; Yamamoto and Inoue, 2024; Zhang Y. et al., 2024). Specifically, great attention has been directed to immune-based and aptamer-based technologies against SARS-COV-2 spike protein (Chen et al., 2023; Dong et al., 2024).

Aptamers are single-stranded oligonucleotides that possess excellent recognition and binding properties to their targets with remarkable affinity (Brown et al., 2024; Mahmoudian et al., 2024). Compared to antibodies, aptamers have advantageous features such as ease of chemical modification, inexpensive production costs, relatively low batch variability, non-immunogenicity, and *in vitro* selection (Alkhamis et al., 2024). Informally known as “chemical antibodies,” aptamers have increasingly become antibody alternatives (Maradani et al., 2024). For the detection of intact SARS-CoV-2 VOCs, aptamers that recognize and bind to accessible surface proteins (e.g., S protein) are necessary (Kacherovsky et al., 2021).

The 150 kDa S protein, a glycosylated homotrimeric class I fusion protein, extends from the viral surface, which plays a pivotal role in viral entry and pathogenesis. Its S1 portion is comprised of the receptor-binding domain (RBD) and N-terminal domain (NTD), while the S2 portion contains the fusion peptide (Cao et al., 2021; Yan et al., 2021; Stäb et al., 2024). Aptamers have been identified against S-protein both as potential drug candidates, e.g., lowering pro-inflammatory response (Kim et al., 2024) and binding inhibition and viral neutralization (Gelinas et al., 2023), and as the main detection elements for diagnostic applications, e.g., point of care detection (Shrikrishna et al., 2024) and environmental monitoring (Yu et al., 2024), among others. However, new mutations have emerged and spread, resulting in unique amino acid profiles among SARS-CoV-2 VOCs. Significantly, numerous alterations in S-protein have undergone positive selection, leading to considerable changes in viral traits, including increased transmissibility and enhanced immune evasion, posing challenges to the current SARS-CoV-2 mitigation approaches (Yao et al., 2024; Yue et al., 2024).

In this study, we aimed to identify and generate aptamer sequences targeting the S-protein of SARS-CoV-2 VOCs through systematic evolution of ligands by exponential enrichment (SELEX). We conducted extensive *in silico* and *in vitro* characterizations to identify robust and specific aptamer sequences. These sequences are potential candidates for addressing SARS-CoV-2 variants.

## 2 Materials and methods

### 2.1 Reagents and materials

Horseradish peroxidase (HRP)-conjugated streptavidin (S-HRP), bovine serum albumin (BSA), Tris-HCl buffer, heparin sodium salt, and clear flat bottom polystyrene high bind 96 microplates (Corning CLS3690) were purchased from Millipore Sigma (St. Louis, MO). Sodium chloride (NaCl), magnesium chloride (MgCl_2_), EDTA, diothiothreitol (DTT), sodium carbonate (Na_2_CO_3_), sodium bicarbonate (NaHCO_3_), phosphate-buffered saline (PBS, 10×), Tween-20, Phusion Flash High-Fidelity PCR Master Mix, Sheared Salmon Sperm DNA, Stop Solution, Dynabeads^™^ His-Tag Isolation and Pulldown, and Influenza A H1N1 HA (A/California/04/2009) were purchased from Thermo Fisher Scientific (Waltham, MA) while 10% Mini-PROTEAN^®^ TBE-Urea Gel was obtained from Biorad (Hercules, CA). TMB One solution was purchased from Promega Corporation (Madison, WI). SARS-CoV-2 recombinant S proteins (40589-V08B1, 40591-V08H23, 40592-V08H12) were obtained from Sino Biological US Inc. (Wayne, PA, United States). Conventional PCR primers, random DNA library pool, and aptamers (regular and modified) developed from this study were synthesized by Integrated DNA Technologies (IDT) (Coralville, IA, United States) (Table 1).

**Table 1:** Sequences of ssDNA library, conventional PCR primers, and aptamers (regular and modified) used and generated in the study.

Name	Sequence
ssDNA Library	5′-ATCGAGCATGGCGAGTGTCC-45N-CGTCTGAATGACGGCTAA CT-3′
VOC Apt-For	5′-ATCGAGCATGGCGAGTGTCC-3′
VOC Apt-Rev	5′-TTTTTTTTTTTTTTTTTTTT/iSp9/AGTTAGCCGTCATTCAGACG-3′
NGS Ext-For	5′-CGTGTGCGTGCTATTAATTGAAATCGGGTAACTTAAATGCATCGAGCATGGCGAGTGTCC-3′
NGS Ext-Rev	5′-TGTAAGTCGCAGTAAGTGGTCCGTATAGTACTCTGAGTCAAGTTAGCCGTCATTCAGACG-3′
Alpha Apt	5′-AGTTAGCCGTCATTCAGACGATTTGTCCGTGTTACGATTGGGGAACGTGGATGACATTCTGGACACTCGCCATGCTCGAT-3′
Delta Apt	5′-ATCGAGCATGGCGAGTGTCCTATCCCCCATCAACTCAACCCAACCAGTACGACTCCTCCTCGTCTGAATGACGGCTAACT-3′
Omicron Apt	5′-CTGGCCTCACTGGATACTCTAAGACTATTGGTCAAGTTTGCCTTGTCAAGGCTATTGGTCAAGGCAAGGCTGGCCAACCCATGGGTGGAGTTTAGCCAG-3′
Alpha Apt (Bio)	5′-/5Biosig-AGTTAGCCGTCATTCAGACGATTTGTCCGTGTTACGATTGG GGAACGTGGATGACATTCTGGACACTCGCCATGCTCGAT-3′
Delta Apt (Bio)	5′-/5Biosig-ATCGAGCATGGCGAGTGTCCTATCCCCCATCAACTCAACCCAACCAGTACGACTCCTCCTCGTCTGAATGACGGCTAACT-3′
Omicron Apt (Bio)	5′-/5Biosig-CTGGCCTCACTGGATACTCTAAGACTATTGGTCAAGTTTGCCTTGTCAAGGCTATTGGTCAAGGCAAGGCTGGCCAACCCATGGGTGGAGTTTAGCCAG-3′

### 2.2 Systematic evolution of ligands by exponential enrichment

**Figure 1:**
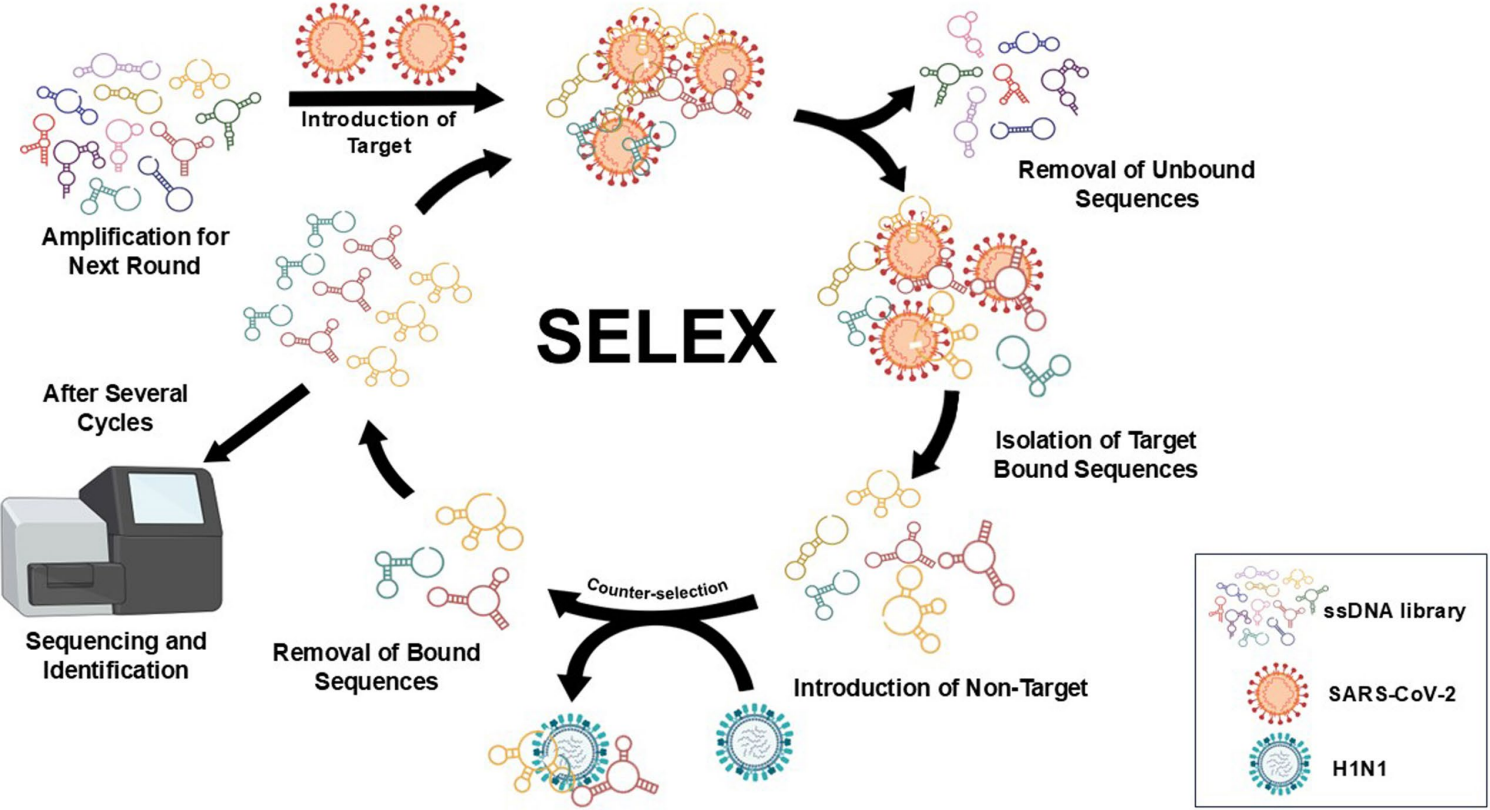
Workflow of SELEX used in the study with minor modifications (Wang et al., 2019). SARS-CoV-2 recombinant S proteins of VOCs on His-Tag Isolation and Pulldown beads were individually introduced to a DNA pool (81-nt) with a 45 random nucleotide region flanked by fixed 3′ and 5′ regions. Unbound ssDNA was discarded using the prepared wash buffer, and the recovered bound ssDNA was used as a template for preparative and amplification of conventional PCR. Products from conventional PCR were pooled and reduced prior to denaturation by TBE-Urea gel. Separated ssDNA bands were viewed using a blue/white light transilluminator to correctly excise the target sense strands. Recovery of ssDNA was achieved by electro-elution of excised bands using dialysis tubes and ethanol precipitation. The procedure was repeated for up to nine rounds; non-target protein (H1N1 HA) was introduced in the fifth round as part of the negative selection process. In the succeeding rounds, the amount of S-protein was gradually reduced, and incubation time with ssDNA pool was shortened while washing time and the amount of random sperm ssDNA both increased. The final products were further amplified using extended primers prior to next-generation sequencing (NGS) and data processing.

An earlier report by Wang et al. (2019) was adapted for SELEX with minor modifications (Figure 1). In this study, S-proteins of each VOC (0.1 nmol) in binding buffer (2.5 mM MgCl_2_, Salmon sperm ssDNA (3 nmol), 0.02% (v/v) Tween-20 and 1 mM heparin in 1 × PBS) were individually immobilized on His-Tag Isolation and Pulldown Beads. A library (100 μM) of DNA sequences (81-nt) with a 45 random nucleotide region flanked by fixed 3′ and 5′ regions was then incubated (1 h) with beads-S-protein complex. Unbound ssDNA was discarded by using the prepared wash buffer (2.5 mM MgCl_2_, 0.02% (v/v) Tween-20 in 1 × PBS). The recovered bound ssDNA was used as a template for preparative and amplification conventional PCR (Supplementary Table S1 and Supplementary Figure S1). Products from conventional PCR were pooled and reduced using a CentriVap micro-IR vacuum concentrator (Labconco, Kansas City, MO) at 3,000 × g (56°C) prior to denaturation by TBE-Urea gel. The reverse primer (VOC Apt—Rev) was modified with polyT (20 × dT) via iSp9 linker to allow the amplification of anti-sense strands with an additional 20-nt on its tail (100-nt), a key feature that would distinguish it from the shorter sense strands (80-nt) during the slicing process.

Separated ssDNA bands were viewed using a blue/white light transilluminator (Thermo Fisher) to correctly excise the target sense strands. Recovery of ssDNA was achieved by electro-elution of excised bands using 3.5 kDa cut-off dialysis tubes (120 V, 20 min) and ethanol precipitation. The procedure was repeated for up to eighth round; non-target protein (H1N1 HA) was introduced in the fifth round as part of the negative selection process. In the succeeding rounds, the amount of S-protein (0.1 nmol to 0.001 nmol) was gradually reduced, and incubation time with ssDNA pool (1 h to 10 min) was shortened while washing time, and the amount of random sperm ssDNA both increased. The final products were further amplified using extended primers prior to shipping to GENEWIZ (Azenta Life Sciences, South Plainfield, NJ, United States) for next-generation sequencing (NGS) and data processing.

### 2.3 Selection and characterization of aptamers

#### 2.3.1 Molecular docking simulation and modeling

**Figure 2:**
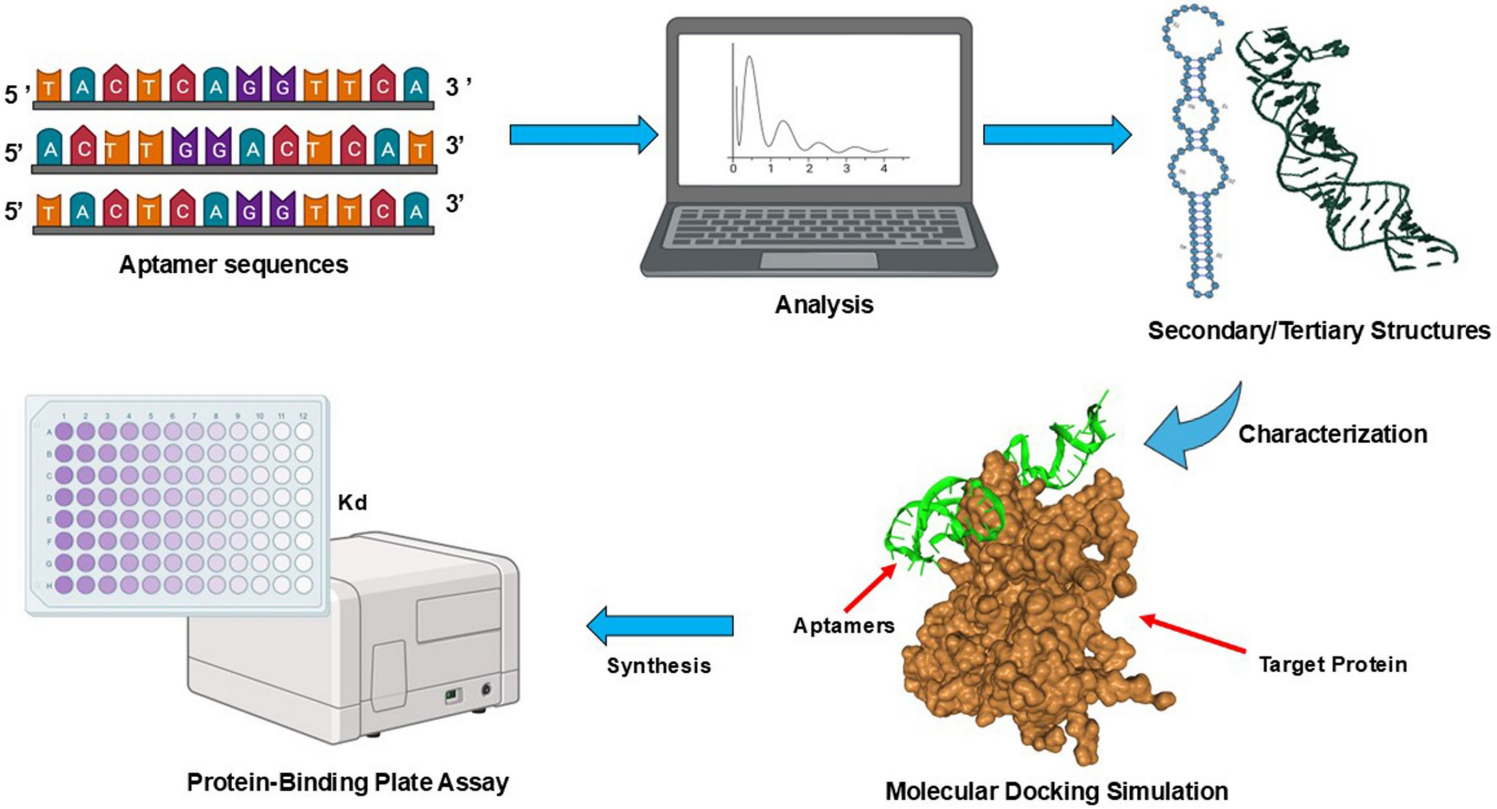
Schematic diagrams of aptamer sequence characterization. Processing workflow from NGS data, predicting secondary and tertiary structures, and molecular docking simulation. A frequency or sequence script was written in Python version 3.12., to analyze the data by clustering and ranking aptamer sequences (https://github.com/Wu-Microbiology/aptamerdocking). The secondary structure prediction was generated and visualized prior to the construction of tertiary structures. The molecular docking simulation of the aptamer and S proteins enabled the ranking of models based on their docking scores. Finally, the binding affinities of SARS-CoV-2 VOCs aptamer candidates were estimated via a direct-ELONA (Pawel et al., 2022).

To process the NGS data, a frequency or sequence script was written in Python version 3.12., which allowed clustering and ranking of sequences in an aptamer pool. The identified sequence was subjected to a simulation workflow, as shown in Figure 2. The secondary structure prediction was generated by RNAsoft CombFold (Andronescu et al., 2003) and visualized using RiboSketch (Lu et al., 2018). Discovery Studio Visualizer 4.0 software (Discovery Studio Visualizer, 2005) and Charmm Gui version 3.8 (Jo et al., 2008) were used for constructing tertiary structures. To perform molecular docking simulation of the aptamer and target protein, the structure of S proteins was downloaded (pdb file) from www.rcsb.org and used as a receptor molecule in HDOCK (Yan et al., 2017) which generated and ranked docking models based on its docking scores. The protein–ligand complex in PDB format generated by HDOCK was then used and uploaded to PLIP version 2.3.0 (Adasme et al., 2021) to determine interaction details of S proteins and aptamer sequence complexes.

#### 2.3.2 Measurement of the SARS-CoV-2 VOCs aptamer dissociation constant (*K*_d_) using direct-enzyme-linked oligonucleotides assay

The binding affinities of SARS-CoV-2 VOCs aptamer candidates were estimated via a direct-enzyme-linked oligonucleotides assay (ELONA) from a previous report with minor modification (Pawel et al., 2022). In brief, a 96-well polystyrene microplate was coated with 1 μg/mL recombinant S proteins (50 μL) for 2 h at room temperature (25°C). The microplate was washed three times with 100 μL PBS-T buffer [1 × PBS with 0.05% (v/v) Tween-20] and blocked with 50 μL PBS-T-BSA [PBS-T with 5% (w/v) BSA] for 1 h at room temperature (25°C). Subsequently, the microplate was washed three times with PBS-T buffer prior to adding increasing concentrations (0–1,000 nM) of biotinylated aptamers (40 μL per concentration, triplicates) in ELONA buffer solution (2 mM MgCl_2_, 0.5 mM CaCl_2_ in 1 × PBS, pH 7.4). The microplate was incubated for another 1 h at room temperature (25°C) and washed three times with PBS-T buffer. A freshly made 1:500 dilution of S-HRP (40 μL) in PBS-T buffer was added to each well for 1 h at room temperature (25°C). Each well was washed three times with PBS-T buffer before adding 50 μL TMB One solution. The microplate was incubated in the dark until the light blue color developed, and the reaction was quenched by adding 10 μL of Stop Solution. The absorbance values at 450 nm were recorded using the BioTek microplate reader. The response values were generated by using Equation 1, where *A* is the absorbance value from each well while *Aɵ* is the absorbance value from the blank. Analysis was performed by calculating statistical parameters and fitting nonlinear curves to determine the *K*_d_ using the Origin software (OriginLab Corp., Northampton, MA, United States).

In brief, a column containing the concentration of aptamer was created in the Origin software (OriginLab Corp., Northampton, MA, United States). Additional columns (triplicates) containing the calculated values from Equation 1 were further included. The mean and standard deviation (S.D.) for each value that corresponded to each concentration or row were generated using the Descriptive Statistics option. The aptamer concentration was plotted against the mean, while the S.D. was set as the error in *Y*. For the fitting option, nonlinear fitting was selected, and the *K*_d_ function was chosen to calculate the *K*_d_ values in nM.


(1) $$\mathrm{Response}=\frac{A-A_{\theta }}{A_{\theta }}$$

## 3 Results

**Figure 3:**
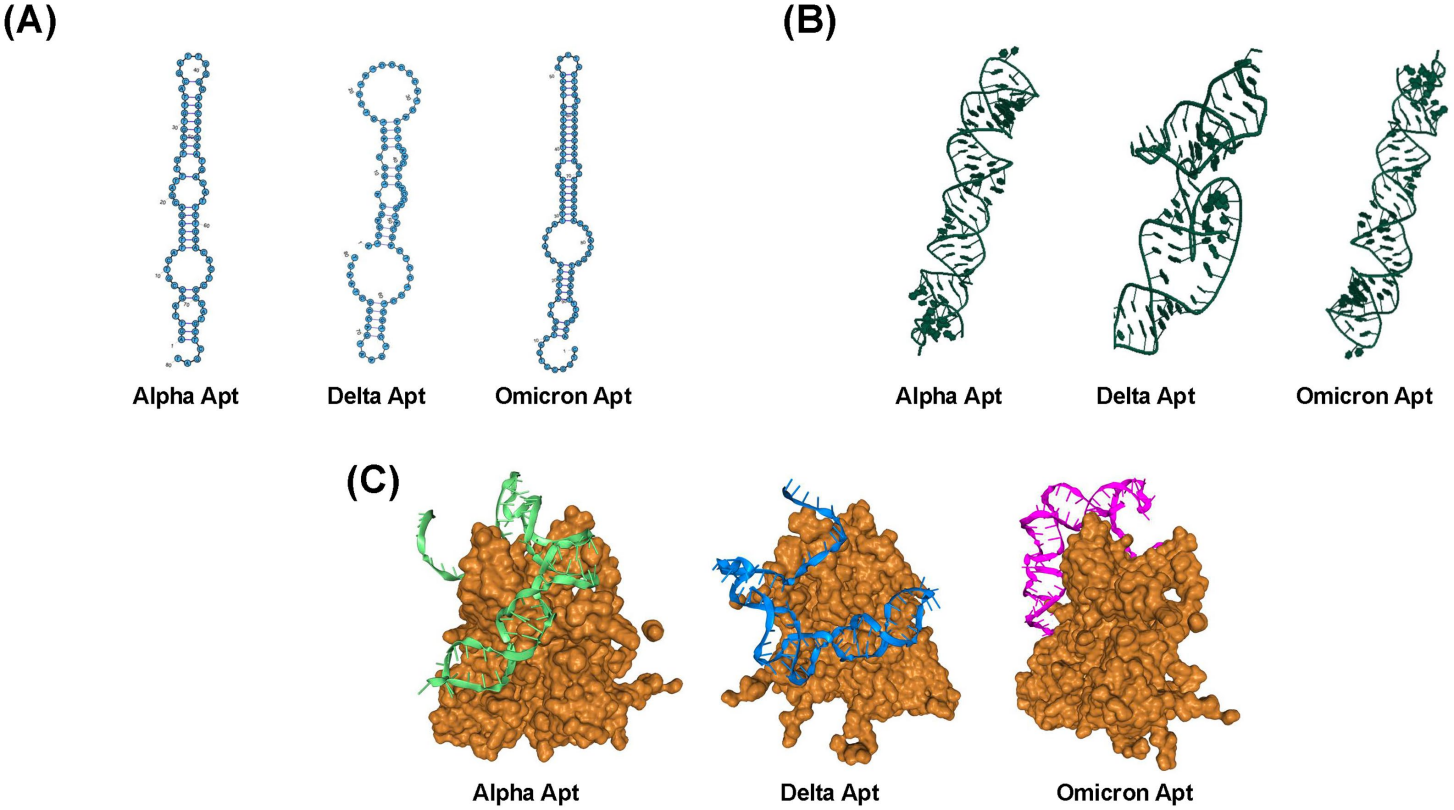
Structures and selection of aptamers from a random library. **(A)** Predicted secondary structures of Alpha Apt, Delta Apt, and Omicron Apt. **(B)** Predicted tertiary structures of Alpha Apt, Delta Apt, and Omicron Apt. **(C)** Top models from molecular docking simulation as predicted by HDOCK (Yan et al., 2017).

In this study, a two-step PCR procedure was conducted per each round of SELEX. The first step (1) preparative PCR was performed to determine the optimum cycle number, while the subsequent step was the amplification PCR (2), where the sub-library was generated. The optimum cycle number was defined as the number of PCR cycles that displayed the highest yield of aptamer fragments while having low amounts of PCR by-products (Supplementary Figure S2). After nine selection cycles, the generated ssDNA pools were processed and sequenced. The most prevalent sequences are as follows: Alpha VOC or Alpha Apt (5′-AGTTAGCCGTCATTCAGACGATTTGTCCGTGTTACGATTGGGGAACGTG GATGACATTCTGGACACTCGCCATGCTCGAT-3′), Delta VOC or Delta Apt (5′-ATCGAGCATGGCGAGTGTCCTATCCCCCATCAACTCAACCCAACCAGTACGACTCCTCCTCGTCTGAATGACGGCTAACT-3′), Omicron VOC or Omicron Apt (5′-CTGGCCTCACTGGATACTCTAAGACTATTGGTCAAGTTTGCCTTGTCAAGGCTATTGGTCAAGGCAAGGCTGGCCAACCCATGGGTGGAGTTTAGCCAG-3′). These sequences were also chosen for further characterization. The most stable secondary structures, i.e., lowest Gibb’s free energy ∆*G*, are shown in Figure 3A, which also indicate the highest thermodynamic stability. Similar to the secondary structure, Figure 3B shows the tertiary structures of the identified aptamer sequences for the three VOCs. Each of these structures has stems and hairpin loops. Molecular docking models are presented in Figure 3C, showing the highest-ranked models based on the docking scores. Supplementary Table S2 summarizes the top eight docking and confidence scores and ligand root-mean-square deviation (RMSD) value, which is the mean distance between the atoms of superimposed molecules (Parisien et al., 2009; Oliveira et al., 2022). Representative macromolecule (S protein)-ligand (Alpha Apt, Delta Apt, and Omicron Apt) complexes’ binding site sections indicate non-covalent interactions such as hydrogen bond, salt bridge, and hydrophobic interactions (Supplementary Figures S3–S5). Details on atomic-level contacts of aptamers, as well as information on amino acid residues, participating ligand/aptamer atoms, and geometry of the interaction (e.g., distance of interacting atoms), are presented in Supplementary Tables S3–S5.

**Figure 4:**
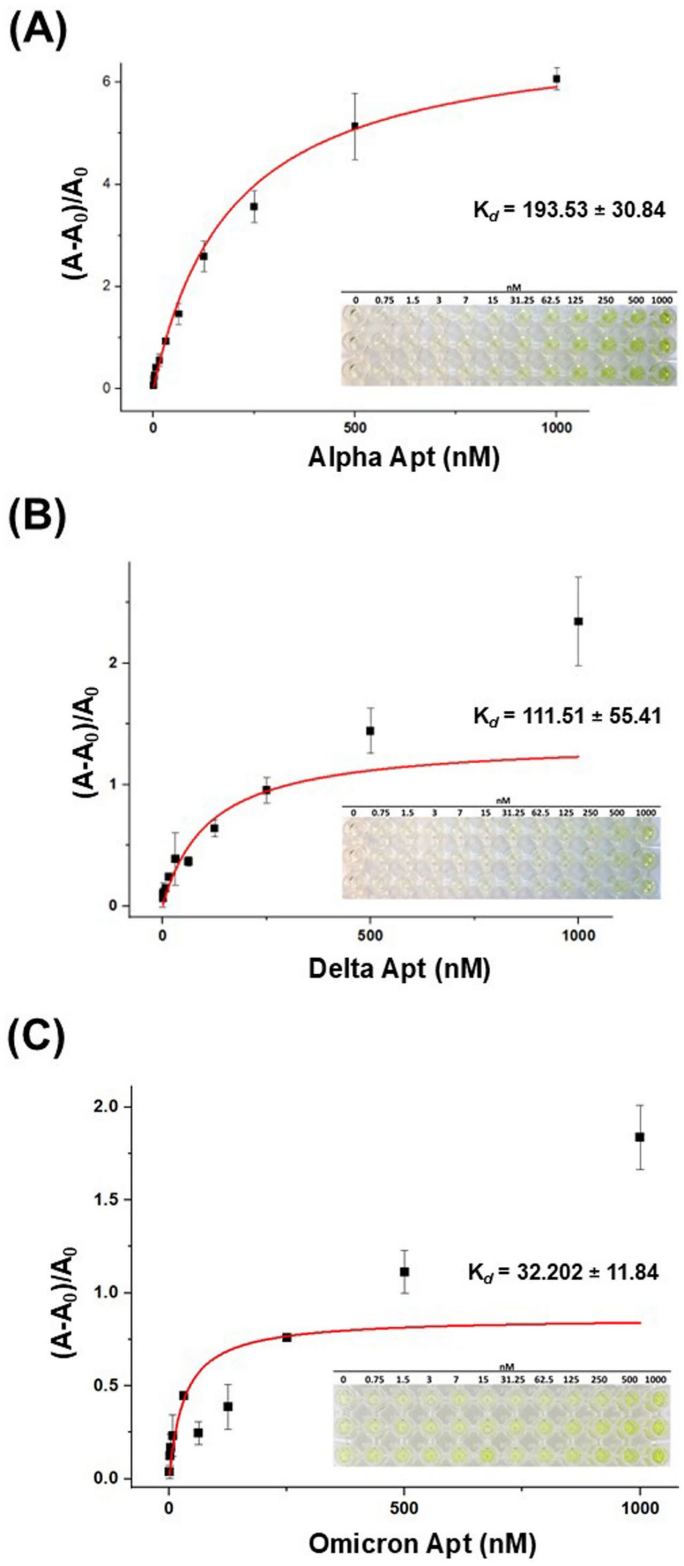
The binding affinity curves of VOC aptamers generated from the ELONA assay. **(A)** The estimated (*K*_d_) value of Alpha Apt (193.53 ± 30.84 nM). **(B)** The estimated (*K*_d_) value of Delta Apt for (111.51 ± 55.41 nM). **(C)** The estimated (*K*_d_) value of Omicron Apt (32 ± 11.84 nM). Insets on the lower right corner of each binding affinity curve are the ELONA reaction microplates showing color reaction gradient. The color intensity decreased as the concentration of aptamers was reduced. *n* = 3 technical replicates, mean ± S.D.

The dissociation constant (*K*_d_) of VOC aptamers was estimated by absorbance using the direct-enzyme-linked oligonucleotides assay (ELONA) method. This approach allowed immobilization of S-protein on a microplate and utilized biotinylated VOC aptamers and S-HRP for a colorimetric reaction to occur via the oxidation of TMB substrate (3,3′,5,5′-tetramethylbenzidine diamine). By adjusting the aptamer concentrations in relation to the fixed concentration of S-protein followed by quantifying the bound aptamer, the binding affinity could be determined as a dissociation constant (*K*_d_). Figures 4A–C show the binding affinity curves of VOC aptamers. The estimated (*K*_d_) values were 193.53 ± 30.84 nM for Alpha Apt, 111.51 ± 55.41 nM for Delta Apt, and 32 ± 11.84 nM for Omicron Apt (Supplementary Figure S3). The S.D., which was less than 60% of the calculated *K*_d_ is typical with this type of affinity estimation (McKeague et al., 2014).

## 4 Discussion

In SELEX, non-specific amplicons or parasite DNA appear to form via primer-primer hybridization and partial binding of primers to the random region of the library, forming products with several base pairs longer than the expected aptamer (Tolle et al., 2014). Unfortunately, due to the reliance of aptamer development on a random library, the amplification of nonspecific sequences appears to be unavoidable once complementary sequences between the primers and library sequences exist. Therefore, selecting a PCR cycle number that both maximizes aptamer amplification while minimizing by-product formation is critical for the efficiency of SELEX. To ensure the high binding of aptamers to the protein target in this study, the concentration of target molecules was reduced with the progression of SELEX to create a more selective SELEX condition. The initial aptamer-to-target ratio used was 50:1 since it has been reported to provide substantial competition between ligands to facilitate the enrichment of high-affinity aptamers (Seo et al., 2010; Liu et al., 2022). The ratio gradually decreased in subsequent rounds of incubation. The poly-T20 with Sp9 bridge allowed denaturing gel electrophoresis-based single-stranded DNA separation (Supplementary Figure S4). Another strategy used in this study for selecting high-binding aptamers was decreasing the incubation time from 1 h in the first round of SELEX to 10 min in the eighth round of SELEX. To stabilize the selection conditions, Mg^2+^ was included in the binding buffer since it has been shown to stabilize the secondary structure of aptamers which results in enhanced binding (Reuss et al., 2014). Additionally, primers were designed to include reasonable annealing temperatures and less than 50% GC content to avoid primer heterodimers and primer self-dimers. The consideration of PCR by-product is especially important since parasite DNA is often the cause of the failure of SELEX (Tolle et al., 2014). By-product formation is generally avoided by lowering the PCR cycle number. This, however, tends to reduce the yield of the desired aptamers. To account for the lower PCR yield, the amplification PCR was conducted to allow larger numbers of PCR reactions. Over time, by-products can still have a large presence in the library. To mitigate this problem, parasite DNA was removed from the library via PAGE gel separation, which allowed the yield of pure desired ssDNA needed for further rounds of SELEX which was completed until round nine.

The NGS data was processed using clustering strategies based on similarities, which identified sequence groups by sequence distribution to aptamer families. Sequence features such as (1) full length, (2) fixed k-mers, and (3) sub-sequences of different lengths can facilitate the clustering of aptamers from sequenced random regions (Sun et al., 2022). In this study, every sequence in the pool was compared and placed in the same cluster based on the similarity threshold. Each representative sequence from clusters was further analyzed, aligned, and truncated. Sequences were filtered even further, which generated the desired aptamer sequences (approx. 80-nt) with the highest frequency. The secondary and tertiary structures of the chosen aptamer sequences are the results of hydrogen bond-based intramolecular base-pairing as determined by structural modeling (Patel et al., 1997; Curtale and Citarella, 2013). For aptamers, the likelihood of its predicted framework relies on the superior flexibility of the deoxyribose-phosphodiester backbone that has six different torsion angles which provide the formation of diversified secondary and tertiary structures (Laing and Schlick, 2011; Navien et al., 2021).

For the molecular docking simulation, the combination of SARS-CoV-2 VOCs aptamers as ligand and S-protein as the receptor was used and evaluated by HDOCK. SARS-CoV-2 VOCs aptamers formed structural motifs (e.g., hairpin loops, stems, and pseudoknots), suggesting that these configurations may have allowed its binding with S-protein via electrostatic interactions, hydrogen bonding, pep stacking or non-covalent interactions between peptides particularly involving aromatic residues or other stacking-prone motifs, hydrophobic interaction, and van der Waals forces or combinations of these various forces (Tan et al., 2016; Navien et al., 2021). The molecular docking scores are estimated by an iterative scoring function of the HDOCK server that allows the ranking of possible binding models based on negative docking score values (Yan et al., 2017). For the ligand RMSD, the values range from 351.8 Å–540.65 Å. The RMSD is a common metric used to assess the quality of protein docking models by quantifying the deviation between the predicted model and a reference structure. It provides an objective and straightforward way to assess the alignment between predicted and reference structures. Lower RMSD values typically suggest that the docked model is more like the reference structure, while higher RMSD values imply greater structural divergence. These values can be highly sensitive to the initial orientation of the docked molecule or the reference structure. Small changes in the pose or the rotation of the ligand (or complex) such as aptamers, may lead to large changes in RMSD, even though the actual binding mode or functional relevance might not have changed significantly. Though the ligand RMSD value is not the most accurate metric for each docking model, it has been used to analyze the structural stability of macromolecules during molecular dynamics simulation analysis (Yan et al., 2017; Hu et al., 2019).

The functionality of aptamers is intrinsically linked to their conformational structure, as their effectiveness relies on the physical compatibility between the oligonucleotide and its target. This structure is determined by the nucleotide sequence and how these nucleotides assemble into a three-dimensional form. Thus, predicting the tertiary structure is essential for pinpointing the residues that dictate the aptamer’s conformation and its interaction with the target. In this study, the presented workflow can successfully simulate molecular docking between VOC aptamers and S-protein, which identifies the physical–chemical interactions. The workflow also functions as a preliminary tool to analyze the potential impact of post-SELEX modifications on aptamer conformation and target interactions. This crucial information will facilitate the identification of residues in various aptamer motifs for potential substitution and will provide a suitable *in silico* platform to guide subsequent laboratory work. While factors other than affinity, such as toxicity, binding specificity, and deliverability, need to be considered in evaluating aptamers, the VOC aptamers—Alpha Apt, Delta Apt, and Omicron Apt presented in this work should certainly be further explored.

The binding of the VOC aptamers to S-protein was assessed using ELONA, wherein the proteins were coated on polystyrene high bind 96 microplates. Previous studies on the adsorption of SARS-CoV-2 spike protein on polystyrene surfaces, such as the one reported by Sahihi and Faraudo (2022), have shown no significant structural changes in the conformation of S-protein. Sahihi and Faraudo (2022) also found that Val and Phe amino acid residues in the receptor binding domain (RBD) have crucial roles in contact initiation to polystyrene surface, and its primary driving forces for adsorption are π–π and hydrophobic interactions of polystyrene with the residues and glycans. Their investigation on RMSD, solvent accessible surface area (SASA), volume of protein, radius of gyration (*R*_g_), and secondary structure of the protein conformations showed that adhesion to the polystyrene surface did not cause any substantial secondary or tertiary structural modifications in the protein conformations. Moreover, a report by Xie et al. (2020) on the characterization of the interaction of SARS-CoV-2 virions with surfaces using an atomic force microscopy (AFM) which measured the adhesion force and energy of the SARS-CoV-2 S-protein with a series of inanimate surfaces including a large diversity of hydrophobic and hydrophilic materials such as metals and glass, fabrics, and plastics found that polystyrene had the strongest adhesion force among the materials tested.

A report on aptamer development for SARS-CoV-2 employed a viro-SELEX method using active SARS-CoV-2 (pseudotyped) with S-protein as the target and negative selection against UV inactivated SARS-CoV-2 (pseudotyped), SARS-CoV-1 (pseudotyped) with S-protein and H5N1 (pseudotyped) generated a 45-nt aptamer with binding constant (*K*_d_) of 79 ± 28 nM (Peinetti et al., 2021). Similarly, Martínez-Roque et al. (2022) utilized a combination of ideal-filter capillary electrophoresis SELEX (IFCE-SELEX), NGS and slot blot binding assay to isolate and validate ssDNA aptamers (aptamer C7; *K*_d_ = 89.41 ± 18 nM and aptamer C9; *K*_d_ = 231.9 ± 15 nM) that can bind to SARS-CoV-2 spike glycoprotein. These previous investigations have generated aptamers with relatively higher binding constants than this study, highlighting the potential benefits of adapting the current methods and technologies to isolate and produce highly specific and stable SARS-CoV-2 aptamers. To minimize the degradation of aptamers over time, they can be protected by conjugation to stabilizing molecules (e.g., PEGylation, nanoparticles), which would prevent their interactions with nucleases and extend their half-life. Binding aptamers to nanoparticles (e.g., gold nanoparticles) or other carrier molecules can also protect them from enzymatic degradation while maintaining their functional properties. In addition, structural modifications like circularization (i.e., joining the 5′ and 3′ ends) can protect aptamers from exonucleases since circular aptamers tend to have increased resistance to degradation. These strategies can be considered in the design of stable aptamers for therapeutic, diagnostic, and research applications.

SARS-CoV-2 continues to evolve, and several newer variants have also emerged. A recombinant lineage of Omicron variant—named XBB, appeared in late 2022, and its descendants have evolved successfully (Zhang Q. E. et al., 2024). The members of the XBB lineage were noted for their improved immune evasion and transmissibility. As new mutations in the spike protein emerge, SELEX libraries can be updated to specifically target these changes, helping to recognize the virus even in the presence of mutations that confer immune escape. Moreover, SELEX can be re-executed using viral particles or synthetic spike protein fragments of these newer variants. By using molecular docking in combination with SELEX, reliable models of newly selected aptamers and their interaction with mutated versions of the spike protein or other viral proteins can be created. These approaches can facilitate the designing of more specific and up-to-date aptamer sequences that could recognize viruses even when mutations arise.

While *in silico* predictions offer a valuable initial step in understanding protein-ligand interactions, they have a few limitations related to the accuracy of structural models, scoring functions, and conformational flexibility. Experimental techniques such as X-ray crystallography, nuclear magnetic resonance (NMR), SPR, isothermal titration calorimetry (ITC), cryo-EM, and mass spectrometry are critical for validating and refining these predictions. A combination of computational and experimental approaches can offer a more comprehensive and reliable understanding of protein-ligand interactions, essential for aptamer discovery and other applications.

This study illustrated that the SELEX procedure was effective in generating in-house aptamers but can also be further optimized with *in silico* techniques by using such as those applied in this work. The molecular docking simulation has been useful in providing a deeper understanding of SARS-CoV-2 VOCs aptamers and S-protein interactions, which is meaningful for the design and optimization of aptamer applications.

## 5 Conclusion

In this report, we presented ssDNA aptamer selection by an *in silico* approach (SELEX) specifically targeting the receptor-binding domain of the S-protein across SARS-CoV-2 VOCs and elucidated the molecular docking and binding dynamics of the identified aptamer sequences against it. The identified aptamer sequences bind to relevant VOCs via the spike proteins with high affinity. The dissociation constant values (*K*_d_) of the aptamers were in the low nM range, while the docking models displayed another layer of understanding of how SARS-CoV-2 VOCs aptamers and S-protein interact at the molecular level, contributing to future antiviral and diagnostic innovation. Future work will be focused on experimental validation of the identified aptamers’ binding affinity and specificity across a broader range of SARS-CoV-2 variants, including new variants that may arise. Further optimization of aptamer stability, selectivity, and performance in diagnostic platforms will be crucial for their clinical application. Additionally, incorporating aptamers into detection systems or therapeutic interventions, such as biosensors or antiviral agents, represents a promising direction. Expanding this research to other viral targets could also contribute to the broader field of aptamer-based diagnostics and therapeutics, paving the way for versatile tools to combat future viral outbreaks.

## Data Availability

The datasets presented in this study can be found in online repositories. The names of the repository/repositories and accession number(s) can be found in the article/Supplementary material.

## References

[ref1] Adasme M. F. Linnemann K. L. Bolz S. N. Kaiser F. Salentin S. Haupt V. J.. (2021). PLIP 2021: expanding the scope of the protein–ligand interaction profiler to DNA and RNA. Nucleic Acids Res. 49, W530–W534. doi: 10.1093/nar/gkab294, PMID: 33950214 PMC8262720

[ref2] Alkhamis O. Canoura J. Wang L. Xiao Y. (2024). Nuclease-assisted selection of slow-off rate aptamers. Sci. Adv. 10:eadl3426. doi: 10.1126/sciadv.adl3426, PMID: 38865469 PMC11168469

[ref3] Andronescu M. Dees D. Slaybaugh L. Zhao Y. Condon A. Cohen B.. (2003). Algorithms for testing that sets of DNA words concatenate without secondary structure. Nat. Comput. 2, 391–415. doi: 10.1023/B:NACO.0000006770.91995.ec

[ref4] Brown A. Brill J. Amini R. Nurmi C. Li Y. (2024). Development of better aptamers: structured library approaches, selection methods, and chemical modifications. Angew. Chem. Int. Ed. 63:e202318665. doi: 10.1002/anie.202318665, PMID: 38253971

[ref5] Cao W. Dong C. Kim S. Hou D. Tai W. Du L.. (2021). Biomechanical characterization of SARS-CoV-2 spike RBD and human ACE2 protein-protein interaction. Biophys. J. 120, 1011–1019. doi: 10.1016/j.bpj.2021.02.007, PMID: 33607086 PMC7886630

[ref6] Chen Y. Zhao X. Zhou H. Zhu H. Jiang S. Wang P. (2023). Broadly neutralizing antibodies to SARS-CoV-2 and other human coronaviruses. Nat. Rev. Immunol. 23, 189–199. doi: 10.1038/s41577-022-00784-3, PMID: 36168054 PMC9514166

[ref7] Curtale G. Citarella F. (2013). Dynamic nature of noncoding RNA regulation of adaptive immune response. Int. J. Mol. Sci. 14, 17347–17377. doi: 10.3390/ijms140917347, PMID: 23975170 PMC3794731

[ref8] Discovery Studio Visualizer. (2005). Accelrys Software Inc. Discovery Studio Visualizer 2.

[ref9] Dong Y. Wang J. Chen L. Chen H. Dang S. Li F. (2024). Aptamer-based assembly systems for SARS-CoV-2 detection and therapeutics. Chem. Soc. Rev. 53, 6830–6859. doi: 10.1039/D3CS00774J, PMID: 38829187

[ref10] Gelinas A. D. Tan T. K. Liu S. Jaramillo J. G. Chadwick J. Harding A. C.. (2023). Broadly neutralizing aptamers to SARS-CoV-2: a diverse panel of modified DNA antiviral agents. Mol. Ther. Nucleic Acids 31, 370–382. doi: 10.1016/j.omtn.2023.01.008, PMID: 36714461 PMC9859636

[ref11] Hu B. Zhou R. Li Z. Ouyang S. Li Z. Hu W.. (2019). Study of the binding mechanism of aptamer to palytoxin by docking and molecular simulation. Sci. Rep. 9:15494. doi: 10.1038/s41598-019-52066-z, PMID: 31664144 PMC6820544

[ref12] Jo S. Kim T. Iyer V. G. Im W. (2008). CHARMM-GUI: a web-based graphical user interface for CHARMM. J. Comput. Chem. 29, 1859–1865. doi: 10.1002/jcc.20945, PMID: 18351591

[ref13] Jung A. Droit L. Febles B. Fronick C. Cook L. Handley S. A.. (2024). Tracking the prevalence and emergence of SARS-CoV-2 variants of concern using a regional genomic surveillance program. Microbiol. Spectr. 12:e0422523. doi: 10.1128/spectrum.04225-23, PMID: 38912809 PMC11302336

[ref14] Kacherovsky N. Yang L. F. Dang H. V. Cheng E. L. Cardle I. I. Walls A. C.. (2021). Discovery and characterization of spike N-terminal domain-binding aptamers for rapid SARS-CoV-2 detection. Angew. Chem. Int. Ed. 60, 21211–21215. doi: 10.1002/anie.202107730, PMID: 34328683 PMC8426805

[ref15] Kim W. Song E. S. Lee S. H. Yang S. H. Cho J. Kim S.-J. (2024). A new DNA aptamer which binds to SARS-CoV-2 spike protein and reduces pro-inflammatory response. Sci. Rep. 14:7516. doi: 10.1038/s41598-024-58315-0, PMID: 38553521 PMC10980804

[ref16] Laing C. Schlick T. (2011). Computational approaches to RNA structure prediction, analysis, and design. Curr. Opin. Struct. Biol. 21, 306–318. doi: 10.1016/j.sbi.2011.03.015, PMID: 21514143 PMC3112238

[ref17] Liu R. Zhang F. Sang Y. Liu M. Shi M. Wang X. (2022). Selection and characterization of DNA aptamers for constructing aptamer-AuNPs colorimetric method for detection of AFM1. Foods 11:1802. doi: 10.3390/foods11121802, PMID: 35742000 PMC9222373

[ref18] Lu J. S. Bindewald E. Kasprzak W. K. Shapiro B. A. (2018). RiboSketch: versatile visualization of multi-stranded RNA and DNA secondary structure. Bioinformatics 34, 4297–4299. doi: 10.1093/bioinformatics/bty468, PMID: 29912310 PMC6289134

[ref19] Lucena R. P. Silva-Junior A. G. Gil L. H. Cordeiro M. T. Andrade C. A. Oliveira M. D. (2024). Application of concanavalin A as a new diagnostic strategy for SARS-COV-2 spike protein. Biochem. Eng. J. 201:109116. doi: 10.1016/j.bej.2023.109116

[ref20] Mahmoudian F. Ahmari A. Shabani S. Sadeghi B. Fahimirad S. Fattahi F. (2024). Aptamers as an approach to targeted cancer therapy. Cancer Cell Int. 24:108. doi: 10.1186/s12935-024-03295-4, PMID: 38493153 PMC10943855

[ref21] Maradani B. S. Parameswaran S. Subramanian K. (2024). Development of DNA aptamers targeting B7H3 by hybrid-SELEX: an alternative to antibodies for immuno-assays. Sci. Rep. 14:13552. doi: 10.1038/s41598-024-64559-7, PMID: 38866941 PMC11169341

[ref22] Marino A. Pampaloni A. Scuderi D. Cosentino F. Moscatt V. Ceccarelli M.. (2020). High-flow nasal cannula oxygenation and tocilizumab administration in patients critically ill with COVID-19: a report of three cases and a literature review. World Acad. Sci. J. 2:1. doi: 10.3892/wasj.2020.6432313883

[ref23] Martínez-Roque M. A. Franco-Urquijo P. A. García-Velásquez V. M. Choukeife M. Mayer G. Molina-Ramirez S. R.. (2022). DNA aptamer selection for SARS-CoV-2 spike glycoprotein detection. Anal. Biochem. 645:114633. doi: 10.1016/j.ab.2022.114633, PMID: 35247355 PMC8889740

[ref24] Mckeague M. Velu R. Hill K. Bardóczy V. Mészáros T. Derosa M. C. (2014). Selection and characterization of a novel DNA aptamer for label-free fluorescence biosensing of ochratoxin a. Toxins 6, 2435–2452. doi: 10.3390/toxins6082435, PMID: 25153252 PMC4147592

[ref25] Meganck R. M. Edwards C. E. Mallory M. L. Lee R. E. Dang H. Bailey A. B.. (2024). SARS-CoV-2 variant of concern fitness and adaptation in primary human airway epithelia. Cell Rep. 43:114076. doi: 10.1016/j.celrep.2024.114076, PMID: 38607917 PMC11165423

[ref26] Navien T. N. Thevendran R. Hamdani H. Y. Tang T.-H. Citartan M. (2021). *In silico* molecular docking in DNA aptamer development. Biochimie 180, 54–67. doi: 10.1016/j.biochi.2020.10.005, PMID: 33086095

[ref27] Nesamari R. Omondi M. A. Baguma R. Höft M. A. Ngomti A. Nkayi A. A.. (2024). Post-pandemic memory T cell response to SARS-CoV-2 is durable, broadly targeted, and cross-reactive to the hypermutated BA. 2.86 variant. Cell Host Microbe 32, 162–169.e3. doi: 10.1016/j.chom.2023.12.003, PMID: 38211583 PMC10901529

[ref28] Oliveira R. Pinho E. Sousa A. L. Dias Ó. Azevedo N. F. Almeida C. (2022). Modelling aptamers with nucleic acid mimics (NAM): from sequence to three-dimensional docking. PLoS One 17:e0264701. doi: 10.1371/journal.pone.0264701, PMID: 35320268 PMC8942228

[ref29] Parisien M. Cruz J. A. Westhof É. Major F. (2009). New metrics for comparing and assessing discrepancies between RNA 3D structures and models. RNA 15, 1875–1885. doi: 10.1261/rna.1700409, PMID: 19710185 PMC2743038

[ref30] Patel D. J. Suri A. K. Jiang F. Jiang L. Fan P. Kumar R. A.. (1997). Structure, recognition and adaptive binding in RNA aptamer complexes. J. Mol. Biol. 272, 645–664. doi: 10.1006/jmbi.1997.1281, PMID: 9368648

[ref31] Pawel G. T. Ma Y. Wu Y. Lu Y. Peinetti A. S. (2022). Binding affinity measurements between DNA aptamers and their virus targets using ELONA and MST. Bio-protoc. 12:e4548. doi: 10.21769/BioProtoc.4548, PMID: 36505027 PMC9709635

[ref32] Peinetti A. S. Lake R. J. Cong W. Cooper L. Wu Y. Ma Y.. (2021). Direct detection of human adenovirus or SARS-CoV-2 with ability to inform infectivity using DNA aptamer-nanopore sensors. Sci. Adv. 7:eabh2848. doi: 10.1126/sciadv.abh2848, PMID: 34550739 PMC8457657

[ref33] Pizzato M. Baraldi C. Boscato Sopetto G. Finozzi D. Gentile C. Gentile M. D.. (2022). SARS-CoV-2 and the host cell: a tale of interactions. Front. Virol. 1:815388. doi: 10.3389/fviro.2021.815388, PMID: 39877344

[ref34] Prabhakaran M. Matassoli F. Leggat D. Hoover A. Srikanth A. Wu W.. (2024). Adjuvanted SARS-CoV-2 spike protein vaccination elicits long-lived plasma cells in nonhuman primates. Sci. Transl. Med. 16:eadd5960. doi: 10.1126/scitranslmed.add5960, PMID: 38170789

[ref35] Reuss A. J. Vogel M. Weigand J. E. Suess B. Wachtveitl J. (2014). Tetracycline determines the conformation of its aptamer at physiological magnesium concentrations. Biophys. J. 107, 2962–2971. doi: 10.1016/j.bpj.2014.11.001 25517161 PMC4269781

[ref36] Sahihi M. Faraudo J. (2022). Molecular dynamics simulations of adsorption of SARS-CoV-2 spike protein on polystyrene surface. J. Chem. Inf. Model. 62, 3814–3824. doi: 10.1021/acs.jcim.2c00562, PMID: 35926227 PMC9364975

[ref37] Seo Y. J. Chen S. Nilsen-Hamilton M. Levine H. A. (2010). A mathematical analysis of multiple-target SELEX. Bull. Math. Biol. 72, 1623–1665. doi: 10.1007/s11538-009-9491-x, PMID: 20077028

[ref38] Shrikrishna N. S. Halder S. Kesarwani V. Nagamani K. Gandhi S. (2024). Unveiling the potential: high-affinity aptamers for point of care detection of SARS-CoV-2 RBD protein and it’s validation in clinical samples. Chem. Eng. J. 493:152841. doi: 10.1016/j.cej.2024.152841, PMID: 39877899

[ref39] Stäb S. Pearce N. M. Tronrud D. E. Ginn H. Fadda E. Santoni G.. (2024). Up, up, down, down: the structural biology of the SARS-CoV-2 spike protein and how it cheats the immune system. Crystallogr. Rev. 30, 74–117. doi: 10.1080/0889311X.2024.2363756, PMID: 39845729

[ref40] Steiner S. Kratzel A. Barut G. T. Lang R. M. Aguiar Moreira E. Thomann L.. (2024). SARS-CoV-2 biology and host interactions. Nat. Rev. Microbiol. 22, 206–225. doi: 10.1038/s41579-023-01003-z 38225365

[ref41] Sun D. Sun M. Zhang J. Lin X. Zhang Y. Lin F.. (2022). Computational tools for aptamer identification and optimization. TrAC Trends Anal. Chem. 157:116767. doi: 10.1016/j.trac.2022.116767

[ref42] Tan S. Y. Acquah C. Sidhu A. Ongkudon C. M. Yon L. Danquah M. K. (2016). SELEX modifications and bioanalytical techniques for aptamer-target binding characterization. Crit. Rev. Anal. Chem. 46, 521–537. doi: 10.1080/10408347.2016.1157014, PMID: 26980177

[ref43] Tolle F. Wilke J. Wengel J. Mayer G. (2014). By-product formation in repetitive PCR amplification of DNA libraries during SELEX. PLoS One 9:e114693. doi: 10.1371/journal.pone.0114693, PMID: 25490402 PMC4260880

[ref44] Wang T. Yin W. Alshamaileh H. Zhang Y. Tran P. H. Nguyen T. N.. (2019). A detailed protein-SELEX protocol allowing visual assessments of individual steps for a high success rate. Hum. Gene Ther. Methods 30, 1–16. doi: 10.1089/hgtb.2018.237, PMID: 30700146

[ref45] Xie L. Liu F. Liu J. Zeng H. (2020). A nanomechanical study on deciphering the stickiness of SARS-CoV-2 on inanimate surfaces. ACS Appl. Mater. Interfaces 12, 58360–58368. doi: 10.1021/acsami.0c16800, PMID: 33337873

[ref46] Yamamoto Y. Inoue T. (2024). Current status and perspectives of therapeutic antibodies targeting the spike protein S2 subunit against SARS-CoV-2. Biol. Pharm. Bull. 47, 917–923. doi: 10.1248/bpb.b23-00639, PMID: 38692869

[ref47] Yan R. Zhang Y. Li Y. Ye F. Guo Y. Xia L.. (2021). Structural basis for the different states of the spike protein of SARS-CoV-2 in complex with ACE2. Cell Res. 31, 717–719. doi: 10.1038/s41422-021-00490-0, PMID: 33737693 PMC7972335

[ref48] Yan Y. Zhang D. Zhou P. Li B. Huang S.-Y. (2017). HDOCK: a web server for protein–protein and protein–DNA/RNA docking based on a hybrid strategy. Nucleic Acids Res. 45, W365–W373. doi: 10.1093/nar/gkx407, PMID: 28521030 PMC5793843

[ref49] Yao Z. Zhang L. Duan Y. Tang X. Lu J. (2024). Molecular insights into the adaptive evolution of SARS-CoV-2 spike protein. J. Infect. 88:106121. doi: 10.1016/j.jinf.2024.106121, PMID: 38367704

[ref50] Yu X. Wang Y. Wang K. Zhu Z. Xiao L. Huang Y.. (2024). Enhanced portable detection for SARS-CoV-2 utilizing DNA tetrahedron-tethered aptamers and a pressure meter. Anal. Methods 16, 639–644. doi: 10.1039/D3AY02100A, PMID: 38205650

[ref51] Yue C. Liu S. Meng B. Fan K. Yang S. Liu P.. (2024). Deletion of V483 in the spike confers evolutionary advantage on SARS-CoV-2 for human adaptation and host-range expansion after a prolonged pandemic. Cell Res. 34, 739–742. doi: 10.1038/s41422-024-01000-8, PMID: 39030295 PMC11442493

[ref52] Zhang Y. Chamblee M. Xu J. Qu P. Shamseldin M. M. Yoo S. J.. (2024). Three SARS-CoV-2 spike protein variants delivered intranasally by measles and mumps vaccines are broadly protective. Nat. Commun. 15:5589. doi: 10.1038/s41467-024-49443-2, PMID: 38961063 PMC11222507

[ref53] Zhang Q. E. Lindenberger J. Parsons R. J. Thakur B. Parks R. Park C. S.. (2024). SARS-CoV-2 omicron XBB lineage spike structures, conformations, antigenicity, and receptor recognition. Mol. Cell 84, 2747–2764.e7. doi: 10.1016/j.molcel.2024.06.028, PMID: 39059371 PMC11366207

