# Supplementary material for: Elucidating the molecular docking and binding dynamics of aptamers with spike proteins across SARS-CoV-2 variants of concern
Source: Front Microbiol. 2025 Feb 14;16:1503890. doi: 10.3389/fmicb.2025.1503890 (PMC11868117; doi:10.3389/fmicb.2025.1503890)
Supplement: Supplementary file 1 [file Data_Sheet_1.pdf]

Supplementary Material

Elucidating the Molecular Docking and Binding Dynamics of Aptamers with Spike Proteins Across SARS-CoV-2 Variants of Concern

Irwin A. Quintela[1], Tyler Vasse[1], Dana Jian[1], Cameron Harrington[1], Wesley Sien[1], and Vivian C.H. Wu[1*]

[1] Produce Safety and Microbiology Research Unit, U.S. Department of Agriculture, Agricultural Research Service, Western Regional Research Center, Albany, CA 94710, USA

*** Correspondence:**
Corresponding Author
vivian.wu@usda.gov

Table S1. SELEX Conventional PCR. Reagents and volumes used for conventional PCR.

Reagents	Reaction mixture (µL)
Forward primer (10 µM)	2.0
Reverse primer (10 µM)	2.0
Phusion Flash High-Fidelity PCR Master Mix	10.5
PCR grade water	Up to 50 µL

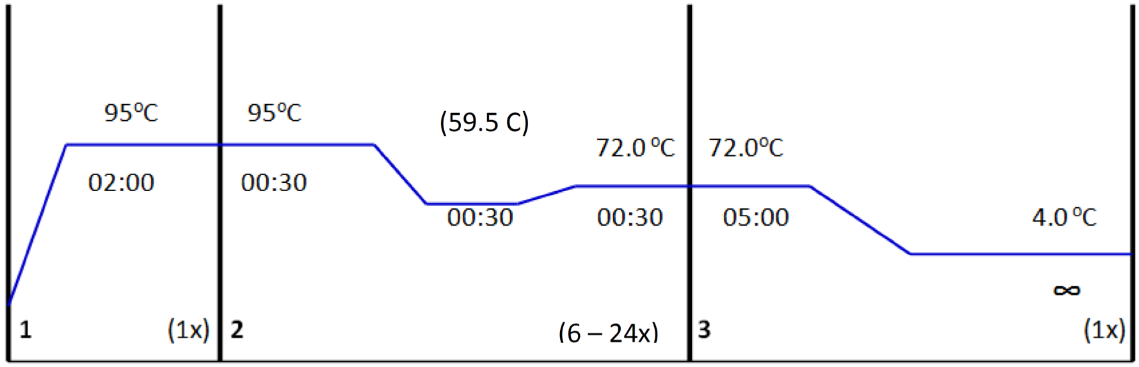

Figure S1. SELEX Conventional PCR conditions. Conditions used to run conventional PCR. Optimal PCR cycles (within 6 – 24x) were determined during preparative PCR.

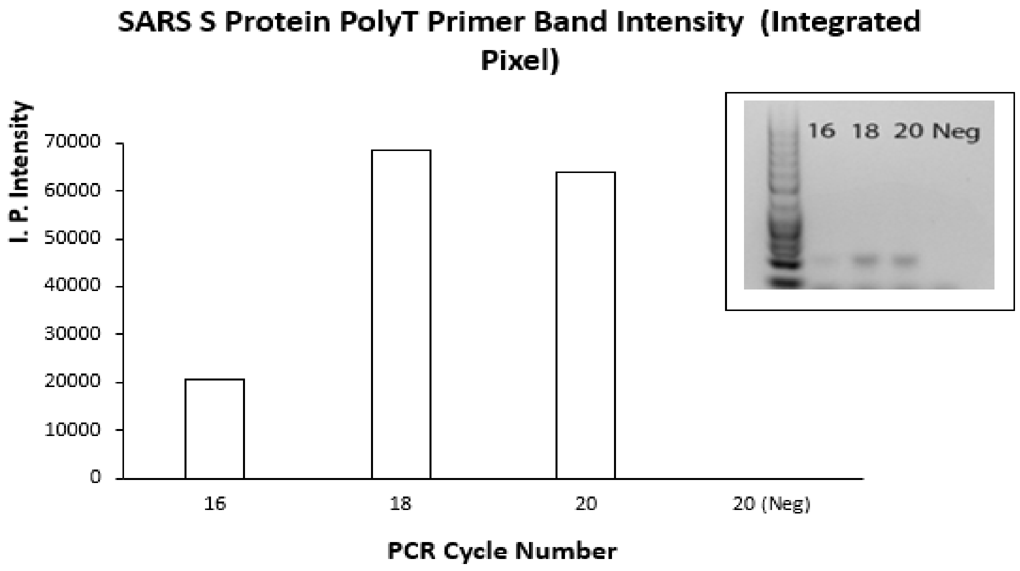

Figure S2. SELEX preparative PCR. The pilot or preparative PCR was conducted to determine the optimum cycle of PCR cycles for the subsequent step, the amplification PCR. The I.P. Intensity showed the highest PCR yield without noticeable non-specific amplicons (inset). In this representative graph, 18 PCR cycles was chosen and used for amplification PCR.

Table S2. **Summary of the top eight molecular docking models using HDOCK** (Yan et al., 2017)). Molecular models were ranked based on individual docking scores.

VOC Aptamers	Rank/Model	Docking Score	Confidence Score	Ligand RMSD (Å)
Alpha Apt	Model 1	-399.26	0.9932	461.87
	Model 2	-385.25	0.9910	399.01
	Model 3	-378.10	0.9897	351.58
	Model 4	-366.51	0.9870	373.93
	Model 5	-365.17	0.9867	393.74
	Model 6	-364.80	0.9866	356.40
	Model 7	-354.54	0.9835	358.38
	Model 8	-351.65	0.9826	360.33
Delta Apt	Model 1	-301.03	0.9535	509.29
	Model 2	-288.30	0.9408	477.88
	Model 3	-283.13	0.9348	521.94
	Model 4	-273.33	0.9218	472.81
	Model 5	-270.90	0.9182	540.65
	Model 6	-263.76	0.9068	491.63
	Model 7	-263.67	0.9066	473.65
	Model 8	-263.07	0.9056	518.16
Omicron Apt	Model 1	-354.22	0.9834	447.51
	Model 2	-347.74	0.9812	488.53
	Model 3	-313.88	0.9637	459.25
	Model 4	-311.85	0.9622	426.29
	Model 5	-295.97	0.9488	448.49
	Model 6	-295.65	0.9485	436.67
	Model 7	-295.33	0.9482	432.68
	Model 8	-295.26	0.9481	458.90

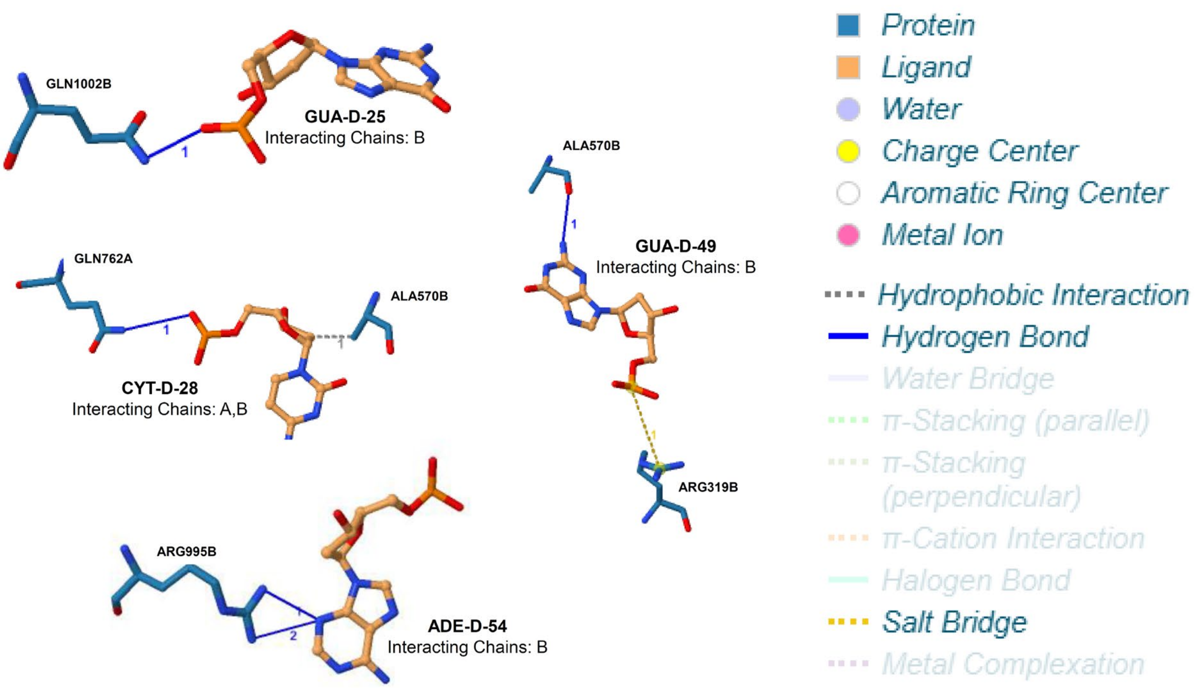

Figure S3. Representative macromolecule (S protein)-ligand complexes showing binding site sections of Alpha Apt and S protein (Adasme et al., 2021). The ligand is shown in orange, and the macromolecule residues are blue. For intra-chain interactions, the chain is shown in orange. Non-covalent interactions are indicated by dashed or solid lines as indicated in the legend on the right side.

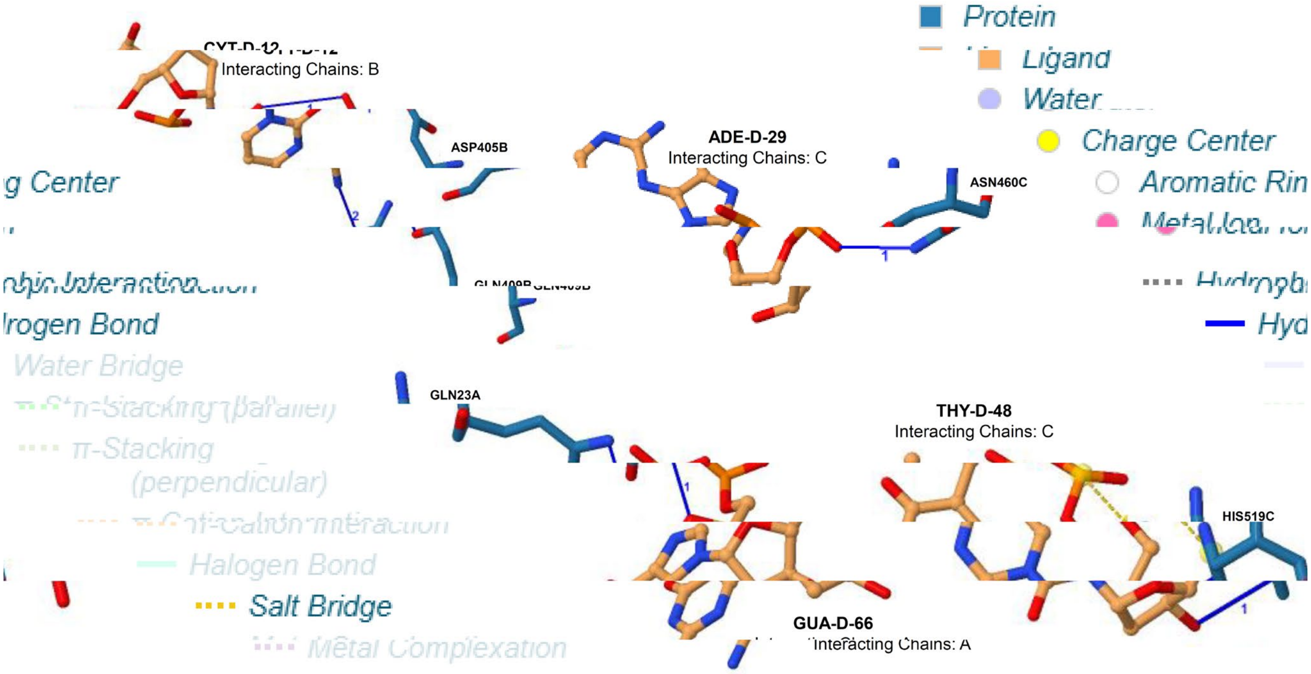

Figure S4. Representative macromolecule (S protein)-ligand complexes showing binding site sections of Delta Apt and S protein (Adasme et al., 2021). The ligand is shown in orange, and the macromolecule residues are shown in blue. For intra-chain interactions, the chain is shown in orange. Non-covalent interactions are indicated by dashed or solid lines as indicated in the legend on the right side

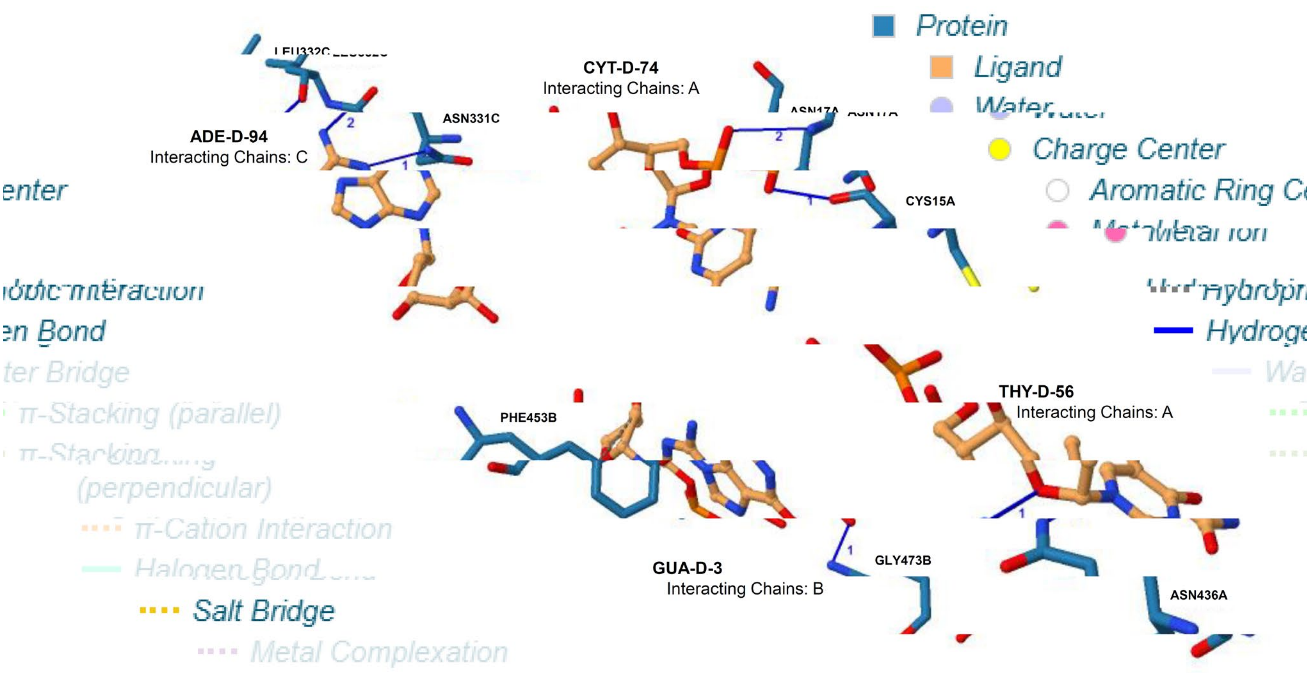

Figure S5. Representative macromolecule (S protein)-ligand complexes showing binding site sections of Omicron Apt and S protein (Adasme et al., 2021). The ligand is shown in orange, and the macromolecule residues are shown in blue. For intra-chain interactions, the chain is shown in orange. Non-covalent interactions are indicated by dashed or solid lines as indicated in the legend on the right side

Table S3. Summary of Alpha Apt contacts in atomic-level detail. Detailed information on amino acid residues, participating ligand/aptamer atoms and geometry of the interaction (e.g. distance of interacting atoms). All atom and residue numbering are in accordance with the numbering in the corresponding PDB file given as input (Adasme et al., 2021).

Small Molecule	Nucleotide	Interacting Chains	Interaction	Residues	Amino Acid	Distance H-A	Distance D-A	Donor Angle	Protein Donor	Side Chain	Donor Atom	Acceptor Atom	Protein Positive	Ligand Group	Ligand Atom	Protein Atom
ADE (Adenine)	ADE-D-18	B	Hydrogen Bond	381B	GLY	2.42	2.91	110.23	Yes	No	9539 [Nam]	15577 [O3]				
	ADE-D-45	B	Hydrogen Bond	1006B	THR	3.44	4.09	126.54	Yes	Yes	13579 [O3]	16136 [O3]				
	ADE-D-54	B	Hydrogen Bond	995B	ARG	2.34	2.95	119.45	Yes	Yes	13494 [Ng+]	16338 [Nar]				
	ADE-D-55	B	Hydrogen Bond	995B	ARG	2.6	3.17	116.38	Yes	Yes	13495 [Ng+]	16338 [Nar]				
	ADE-D-56	B	Hydrogen Bond	992B	GLN	3.44	3.9	110.72	Yes	Yes	13468 [Nam]	16365 [O2]				
CYT (Cytosine)	CYT-D-11	A	Hydrogen Bond	429A	PHE	2.98	3.68	128.32	Yes	No	2633 [Nam]	15435 [O2]				
	CYT-D-28	A	Hydrogen Bond	762A	GLN	3.28	3.81	115.3	Yes	Yes	4548 [Nam]	15781 [O2]				
	CYT-D-28	B	Hydrophobic Interaction	570B	ALA	2.34									15796	10733
GUA (glutaric acid)	GUA-D-20	B	Salt Bridge	386B	LYS	5.31							Yes	Phosphate	15616, 15616, 15619, 15617, 15618	
	GUA-D-25	B	Hydrogen Bond	1002B	GLN	2	2.77	133.33	Yes	Yes	13550 [Nam]	15720 [O2]				
	GUA-D-31	B	Hydrogen Bond	317B	ASN	3.09	4.01	155.02	No	Yes	15851 [Npl]9050 [O2]					
	GUA-D-43	A	Hydrogen Bond	1008A	VAL	3.47	4.06	121.75	No	No	16093 [O3]6233 [O2]					
	GUA-D-43	B	Hydrophobic Interaction	1013B	ILE	3.76									16111	13640
	GUA-D-47	B	Hydrogen Bond	317B	ASN	3	3.42	106.86	Yes	Yes	9051 [Nam]	16181 [O3]				
	GUA-D-49	B	Hydrogen Bond	570B	ALA	2.8	3.13	100.13	No	No	16227 [Npl]	10732 [O2]				
	GUA-D-50	B	Salt Bridge	319B	ARG	5.26							Yes	Phosphate	16217, 16217, 16220, 16219, 16218	
THY (TDP-COI, CID11840985, AGN-PC-00H9BE)	THY-D-22	B	Hydrogen Bond	969B	ASN	2.76	3.63	147.73	Yes	Yes	13295 [Nam]	15670 [O2]				
	THY-D-23	B	Hydrogen Bond	995B	ARG	3.5	4.05	117.74	Yes	Yes	13495 [Ng+]	15690 [O2]				
	THY-D-24	B	Hydrogen Bond	968B	SER	3.31	3.74	109.2	Yes	Yes	13287 [O3]	15700 [O2]				
	THY-D-32	B	Hydrogen Bond	613B	GLN	2.02	2.93	153.8	Yes	Yes	11050 [Nam]15882 [O3]					

	THY-D-48	B	Hydrogen Bond	319B	ARG	3	3.97	170.11	Yes	Yes	9072 [Ng+]	16198 [O2]

Table S4. Summary of Delta Apt contacts in atomic-level detail. Detailed information on amino acid residues, participating ligand/aptamer atoms and geometry of the interaction (e.g. distance of interacting atoms). All atom and residue numbering are in accordance with the numbering in the corresponding PDB file given as input (Adasme et al., 2021).

Small Molecule	Nucleotide	Interacting Chains	Interaction	Residues	Amino Acid	Distance H-A	Distance D-A	Donor Angle	Protein Donor	Side Chain	Donor Atom	Acceptor Atom	Protein Positive	Ligand Group	Ligand Atom	Protein Atom
ADE (Adenine)	ADE-D-29	C	Hydrogen Bond	460C	ASN	3.03	3.58	116.5	Yes	Yes	11859 [Nam]	25643 [O2]				
	ADE-D-33	A	Hydrophobic Interaction	376A	THR	3.58									25740	2800
	ADE-D-38	B	Hydrogen Bond	995B	ARG	3.2	4.08	148.44	Yes	Yes	23888 [Ng+]	25834 [Nar]				
	ADE-D-46	C	Hydrogen Bond	514C	SER	3.44	3.95	114.89	No	Yes	25997 [Npl]	12287 [O3]				
	ADE-D-49	C	Salt Bridge	519C	HIS	4.74							Yes	Phosphate	26043, 26043, 26044, 26046, 26045	
	ADE-D-77	A	Salt Bridge	529A	LYS	4.97							Yes	Phosphate	26610, 26610, 26612, 26613, 26611	
CYT (Cytosine)	CYT-D-12	B	Hydrogen Bond	405B	ASP	3.54	4.03	114.85	Yes	Yes	19709 [O3]	25313 [O2]				
	CYT-D-13	B	Hydrogen Bond	409B	GLN	2.83	3.35	113.39	No	Yes	25316 [Npl]	19744 [O2]				
	CYT-D-19	A	Hydrogen Bond	440A	ASN	2.99	3.81	142.5	Yes	Yes	3303 [Nam]	25453 [O3]				
	CYT-D-39	A	Hydrogen Bond	988A	GLU	2.36	3.24	157.21	Yes	Yes	7140 [O3]	25856 [Nar]				
	CYT-D-39	B	Hydrogen Bond	988A	GLU	2.36	3.24	157.21	Yes	Yes	7140 [O3]	25856 [Nar]				
	CYT-D-40	B	Hydrophobic Interaction	414B	GLN	3.94									25872	19775
	CYT-D-40	B	Hydrogen Bond	408B	ARG	3.06	3.62	117.49	Yes	Yes	19735 [Ng+]	25874 [O2]				
	CYT-D-45	C	Hydrogen Bond	427C	ASP	3.03	3.84	146.99	Yes	Yes	11585 [O3]	25973 [O2]				
GUA (glutaric acid)	GUA-D-17	A	Hydrogen Bond	374A	PHE	2.93	3.88	161.69	Yes	No	2777 [Nam]	25426 [O3]				
	GUA-D-47	C	Hydrogen Bond	519C	HIS	2.87	3.4	114.89	Yes	Yes	12330 [Npl]	26022 [O3]				
	GUA-D-66	A	Hydrogen Bond	23A	GLN	2.1	2.8	126.01	Yes	Yes	83 [Nam]	26387 [O3]				
	GUA-D-70	A	Hydrogen Bond	87A	ASN	2.97	3.41	108.56	Yes	Yes	564 [Nam]	26467 [O3]				

Small Molecule	Nucleotide	Interacting Chains	Interaction	Residues	Amino Acid	Distance H-A	Distance D-A	Donor Angle	Protein Donor	Side Chain	Donor Atom	Acceptor Atom	Protein Positive	Ligand Group	Ligand Atom	Protein Atom
THY (TDP-COI, CID11840985, AGN-PC-00H9BE)	THY-D-18	A	Hydrogen Bond	374A	PHE	2.8	3.37	117.97	Yes	No	2777 [Nam]	25428 [O3]				
	THY-D-48	C	Hydrogen Bond	519C	HIS	3.45	3.96	114.56	Yes	Yes	12333 [Npl]	26042 [O3]				
	THY-D-48	C	Salt Bridge	519C	HIS	4.02							Yes	Phosphate	26023, 26023, 26026, 26024, 26025	
	THY-D-76	A	Hydrophobic Interaction	531A	THR	3.66									26607	4029

Table S5. Summary of Omicron Apt contacts in atomic-level detail. Detailed information on amino acid residues, participating ligand/aptamer atoms and geometry of the interaction (e.g. distance of interacting atoms). All atom and residue numbering are in accordance with the numbering in the corresponding PDB file given as input (Adasme et al., 2021).

Small Molecule	Nucleotide	Interacting Chains	Interaction	Residues	Amino Acid	Distance H-A	Distance D-A	Donor Angle	Protein Donor	Side Chain	Donor Atom	Acceptor Atom	Protein Positive	Ligand Group	Ligand Atom	Protein Atom
ADE (Adenine)	ADE-D-49	A	Hydrogen Bond	343A	ARG	3.28	4.06	137.63	Yes	Yes	2627 [Ng+]	27583 [O3]				
	ADE-D-94	C	Hydrogen Bond	331C	ASN	2.56	3.3	131.78	Yes	Yes	19820 [Nam]	28505 [Nar]				
	ADE-D-94	C	Hydrogen Bond	332C	LEU	2.22	3.17	160.29	No	No	28510 [Npl]	19824 [O2]				
CYT (Cytosine)	CYT-D-1	B	Hydrogen Bond	471B	GLN	2.91	3.88	172.29	No	No	26600 [Npl]	12395 [O2]				
	CYT-D-25	A	Hydrogen Bond	153A	ARG	3.19	3.62	108.48	Yes	Yes	1112 [Ng+]	27075 [O2]				
	CYT-D-60	C	Hydrogen Bond	483C	PHE	2.92	3.47	116.38	Yes	No	21032 [Nam]	27793 [O2]				
	CYT-D-74	A	Hydrogen Bond	15A	CYS	1.9	2.86	168.84	No	No	28087 [O3]	13 [O2]				
	CYT-D-74	A	Hydrogen Bond	17A	ASN	2.67	3.55	148.41	Yes	No	23 [Nam]	28086 [O2]				
	CYT-D-96	A	Hydrogen Bond	164A	GLU	3.44	3.87	110.24	Yes	Yes	1196 [O3]	28537 [O3]				
	CYT-D-96	A	Hydrogen Bond	164A	GLU	3.22	3.87	126.03	No	Yes	28537 [O3]	1196 [O3]				
GUA (glutaric acid)	GUA-D-3	B	Hydrophobic Interaction	453B	PHE	3.64									26643	12243
	GUA-D-3	B	Hydrogen Bond	73B	GLY	2.06	2.52	106.66	Yes	No	12406 [Nam]	26639 [O2]				
	GUA-D-4	B	Hydrogen Bond	486B	TYR	1.93	2.81	155.61	Yes	Yes	12504 [O3]	26657 [Nar]				
	GUA-D-50	A	Salt Bridge	343A	ARG	5.08							Yes	Phosphate	27584, 27584, 27586,	

															27585, 27587	
	GUA-D-57	A	Hydrogen Bond	434A	ASN	3.37	3.9	116.05	Yes	Yes	3357 [Nam]	27734 [O3]				
	GUA-D-57	A	Hydrogen Bond	437A	LYS	3.49	4.07	119.37	No	Yes	27738 [Npl]	3380 [N3]				
	GUA-D-73	C	Hydrogen Bond	15A	CYS	3.31	4.07	135.27	Yes	No	10 [Nam]	28065 [O2]				
	GUA-D-73	C	Hydrogen Bond	17A	ASN	3.26	3.97	130.09	Yes	Yes	30 [Nam]	28069 [O3]				
	GUA-D-73	C	Hydrogen Bond	17A	ASN	2.83	3.5	125.84	Yes	No	23 [Nam]	28084 [O3]				
	GUA-D-90	A	Salt Bridge	127A	LYS	5.47							Yes	Phosphate	28414, 28414, 28417, 28415, 28416	
	GUA-D-95	C	Salt Bridge	337C	GLU	4.49							No	Guanidine	28525, 28524, 28527	
THY (TDP-COI, CID11840985, AGN-PC-00H9BE)	THY-D-2	B	Hydrogen Bond	418B	TYR	3.41	4.03	126.01	Yes	Yes	11953 [O3]	26623 [O3]				
	THY-D-2	B	Hydrogen Bond	471B	GLN	2.98	3.91	157.07	No	No	26616 [Nar]	12395 [O2]				
	THY-D-2	B	Hydrogen Bond	473B	GLY	3.2	3.55	102.47	Yes	No	12406 [Nam]	26618 [O2]				
	THY-D-26	A	Hydrogen Bond	14A	GLN	2.68	3.64	166.35	Yes	Yes	9 [Nam]	27094 [O2]				
	THY-D-56	A	Hydrogen Bond	436A	ASN	2.43	3.24	139.45	Yes	Yes	3371 [Nam]	27714 [O3]				
	HY-D-59	A	Hydrophobic Interaction	372A	PHE	3.7									27788	2870
	THY-D-71	A	Hydrogen Bond	132A	GLN	2.99	3.88	151.31	Yes	Yes	927 [Nam]	28040 [O3]				

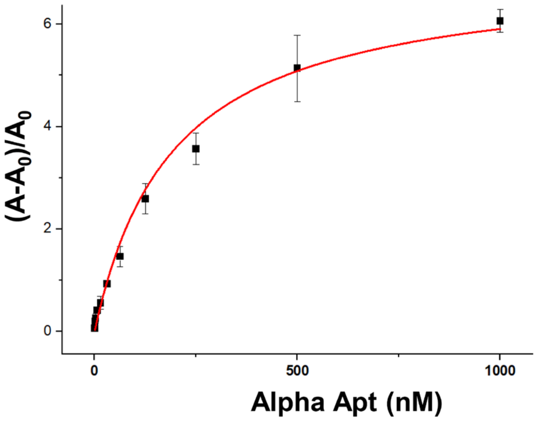

Model	Hyperbl
Equation	y = P1*x/(P2 + x)
Plot	Mean
P1	7.05337 ± 0.62117
P2	193.53527 ± 30.84666
Reduced Chi-Sqr	3.92605
R-Square (COD)	0.96554
Adj. R-Square	0.96171

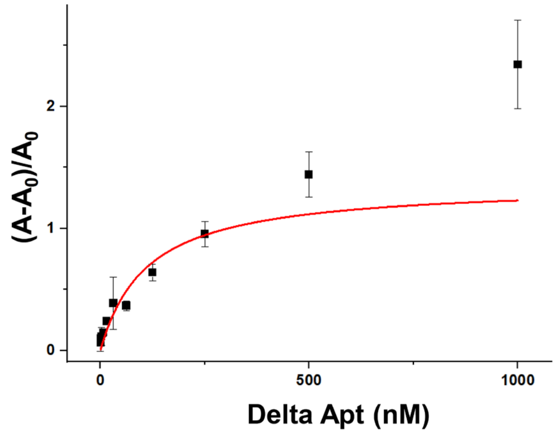

Model	Hyperbl
Equation	y = P1*x/(P2 + x)
Plot	Mean
P1	1.3666 ± 0.38649
P2	111.51745 ± 55.418
Reduced Chi-Sqr	5.22008
R-Square (COD)	0.75849
Adj. R-Square	0.73166

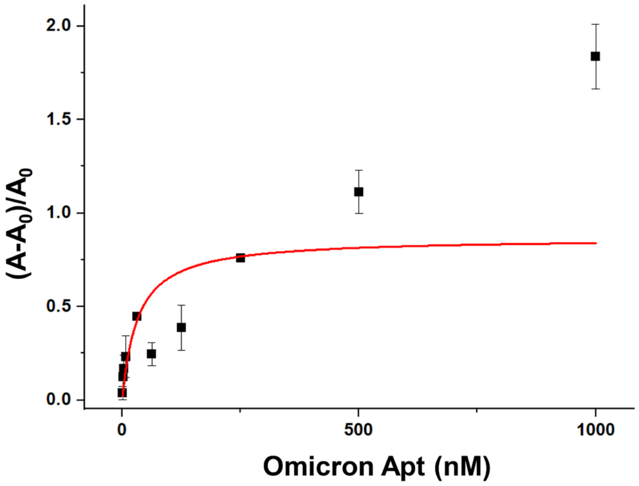

Model	Hyperbl
Equation	y = P1*x/(P2 + x)
Plot	Mean
P1	0.86685 ± 0.09367
P2	32.20248 ± 11.84248
Reduced Chi-Sqr	10.17822
R-Square (COD)	0.84827
Adj. R-Square	0.8293

Figure S3. Analysis of ELONA data using the Origin Software . Each graph shows equation and parameters to generate Kd values (nM).

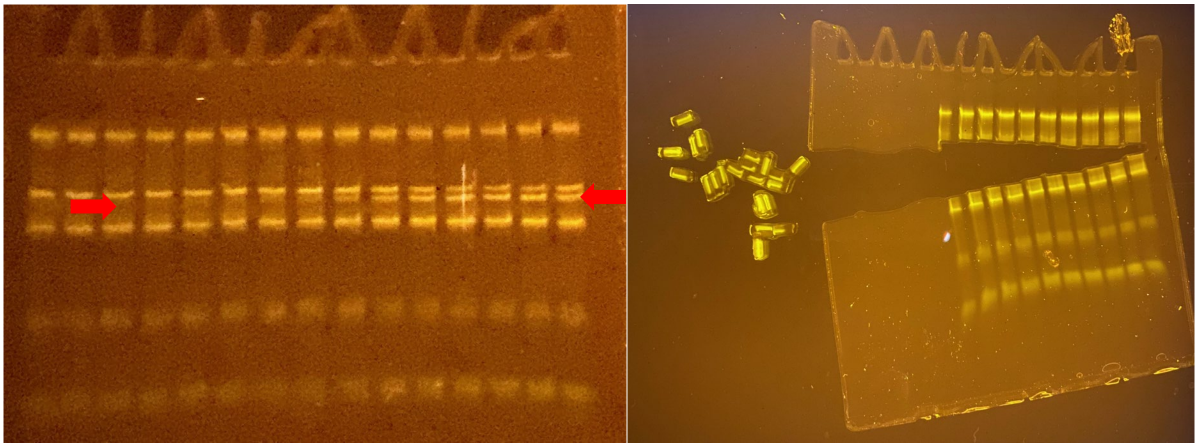

Figure S4. Denaturation of dsDNA using PAGE gel electrophoresis. Under a Blue light trans-illuminator, bands were excised to isolate single stranded DNA during SELEX rounds. The antisense primer with poly-T20 allowed the separation of DNA (lower bands).

References

Adasme, M.F., Linnemann, K.L., Bolz, S.N., Kaiser, F., Salentin, S., Haupt, V.J., and Schroeder, M. (2021). PLIP 2021: Expanding the scope of the protein–ligand interaction profiler to DNA and RNA. *Nucleic acids research* 49**,** W530-W534.

Yan, Y., Zhang, D., Zhou, P., Li, B., and Huang, S.-Y. (2017). HDOCK: a web server for protein–protein and protein–DNA/RNA docking based on a hybrid strategy. *Nucleic acids research* 45**,** W365-W373.
